# The essential roles of small non-coding RNAs and RNA modifications in normal and malignant hematopoiesis

**DOI:** 10.3389/fmolb.2023.1176416

**Published:** 2023-03-31

**Authors:** Xinyi Cai, Hui Wang, Yingli Han, He Huang, Pengxu Qian

**Affiliations:** ^1^ Center for Stem Cell and Regenerative Medicine and Bone Marrow Transplantation Center of the First Affiliated Hospital, Zhejiang University School of Medicine, Hangzhou, China; ^2^ Liangzhu Laboratory, Zhejiang University Medical Center, Hangzhou, China; ^3^ Institute of Hematology, Zhejiang University & Zhejiang Engineering Laboratory for Stem Cell and Immunotherapy, Hangzhou, China; ^4^ Bone Marrow Transplantation Center, The First Affiliated Hospital, Zhejiang University School of Medicine, Hangzhou, China

**Keywords:** hematopoietic stem cell, small non-coding RNAs, RNA modification, hematological malignances, epigenetic

## Abstract

Hematopoietic stem cells (HSCs) developing from mesoderm during embryogenesis are important for the blood circulatory system and immune system. Many factors such as genetic factors, chemical exposure, physical radiation, and viral infection, can lead to the dysfunction of HSCs. Hematological malignancies (involving leukemia, lymphoma, and myeloma) were diagnosed in more than 1.3 million people globally in 2021, taking up 7% of total newly-diagnosed cancer patients. Although many treatments like chemotherapy, bone marrow transplantation, and stem cell transplantation have been applied in clinical therapeutics, the average 5-year survival rate for leukemia, lymphoma, and myeloma is about 65%, 72%, and 54% respectively. Small non-coding RNAs play key roles in a variety of biological processes, including cell division and proliferation, immunological response and cell death. With the development of technologies in high-throughput sequencing and bioinformatic analysis, there is emerging research about modifications on small non-coding RNAs, as well as their functions in hematopoiesis and related diseases. In this study, we summarize the updated information of small non-coding RNAs and RNA modifications in normal and malignant hematopoiesis, which sheds lights into the future application of HSCs into the treatment of blood diseases.

## 1 HSCs support the whole hematopoietic system

Hematopoietic stem cells (HSCs) are one type of pluripotent stem cells that mainly exists in the bone marrow, circulating blood, and cord blood. Since their first discovery by Till and McCulloch in 1961 from the mouse spleens (Till and McCulloch, 2011), research on HSCs has come a long way. The most creative and valuable technologies for HSC study are cell purification and clonal assays. Based on these technologies, scientists can trace HSCs from their origins in early embryos to the terminally differentiated mature cells, as well as a series of physiological processes. Markers in HSC research, usually the cell surface antigens, have been widely used to identify the subsets of HSCs and differentiated downstream progenitors and mature hematopoietic cells (Trusler et al., 2018). CD34, a highly glycosylated cell surface antigen, was the first molecule extensively studied in the isolation and identification of hematopoietic stem cells and their progenitor cells (Slukvin et al., 2022).

There are three types of cell division for HSCs: symmetric self-renewal with products of two HSCs, symmetric differentiation with products of two progenitor cells (HPCs), and asymmetric self-renewal with products of one HSC and one HPC. The HSC niche, a classical concept about the location and microenvironment of HSCs, has been verified to function in the HSC self-renewal regulation with the cooperation of various types of cells (osteoblasts, stromal cells, endothelial cells, megakaryocytes, macrophages, etc.) and cytokines (SCF, CXCL12, TGFβ, FGF1, etc.) (Pinho and Frenette, 2019). It is noticeable that significant differences exist in self-renewal ability between fetal and adult HSCs. Several extrinsic and intrinsic regulators are specially expressed in fetal HSCs, such as the GPI-80 contributing to HSC expansion and the enhancer of zeste homolog 2 (EZH2) functioning in the epigenetic regulation of HSCs (Prashad et al., 2015).

Recent studies have emerged that multiple lineages have already existed in HSCs and HPCs, indicating direct fate-deciding lineage from different HSC subsets to distinct blood cell types (Notta et al., 2016); (Carrelha et al., 2018). Although the exact mechanism remains unclear, environmental stimulation and epigenetic regulation that activate the gene module are regarded as important factors in deciding the direction of downstream differentiation (Wilkinson et al., 2020). The HSC niche has been verified to control the HSC differentiation through extensive intercellular communications. For example, IL-7 or IL-7Rα deficient mice suffer from 10- to 100- fold reduction of B and T lymphocytes, indicating IL-7/IL-7R signaling-dependent lymphoid differentiation of HSC (Carvalho et al., 2001).

HSC trafficking is another essential biological process for both embryonic development and adult hematopoiesis, involving migration, homing, and engraftment. Usually, HSCs travel from one niche to another following active navigation and finally engraft at that position. Relevant research is of great significance to clinical hematological and tumor diseases, which can increase the rate of treatment success of allogeneic hematopoietic stem cell transplantation. Besides, vascular architecture and usher cells were found to regulate and direct HSPC retention, and VCAM-1+ macrophages guide the homing of HSPCs to a vascular niche (Li et al., 2018). As a distinct population, bone marrow dendritic cells control HSPC trafficking, which has been proven partially *via* sinusoidal CXCR2 signaling and vascular permeability (Zhang et al., 2019).

Although HSCs have the self-renew capability, it is inevitable that the HSC aging happens during organismal aging, ultimately exacerbating this process. Progressive telomerase loss, niche changes, and DNA damage accumulation cause functional decline as well as the myeloid bias of aged HSCs. Many hematological dysfunctions and pathological processes, such as leukemia, lymphoid deficiency, and myelodysplastic syndrome, are related to aging of HSCs (Chung and Park, 2017); (Mejia-Ramirez and Florian, 2020). A recent research revealed that loss of autophagy in HSCs leads to mitochondrial aggregation and an activated metabolic state, which reduces the ability of HSCs to self-renew and regenerate (Ho et al., 2017). It is also reported that about 1/3 of aged mitochondria have high autophagy levels in old mice, indicating the possibility of potent HSCs remaining and dilution by less potent cells in the aged mice (Ho et al., 2017). Another recent study found that SIRT1 knockout can prevent age-dependent mixed phenotype acute leukemia (MPAL) and HSC aging, suggesting a potential future avenue for treating MPAL and modifying the functions of aging HSCs (Wang et al., 2022).

## 2 Small non-coding RNAs in normal hematopoiesis and relevant diseases

Non-coding RNAs account for more than 95% of the total genome transcripts and play important roles in regulating various physiological processes (Mattick, 2001), including growth and development, gene silencing, virus resistance, etc. According to its length, non-coding RNA can be divided into long non-coding RNA and small non-coding RNA. In this review, we mainly summarize the recent findings of small non-coding RNAs.

### 2.1 miRNAs

MicroRNAs (miRNAs) are one type of small non-coding RNA usually with 18–24 nucleotides (Sun et al., 2010). There are two sources of miRNAs: one is the transcription product of independent genes, and the other is the intron part of protein-coding genes. The process of miRNA production includes the original transcription, intranuclear hairpin structure formation and processing, transportation through the nuclear pore complex, and the final binding to target mRNAs (Wilson and Doudna, 2013). Mature miRNAs can either lead to the degradation of target mRNA or inhibition of mRNA translation with the aid of RISC complex (Fabian and Sonenberg, 2012). In these cases, miRNAs regulate a variety of physiological processes in eukaryotic organisms. Meanwhile, miRNAs are also regulated by many regulatory pathways. Autoregulatory feedback loop functions in miRNA gene transcription and many co-operators of miRNAs, such as Drosha and Dicer, as well as small molecules contribute to the regulation of miRNA expression (Krol et al., 2010). Recent studies revealed that protein-coding genes also participate in miRNA regulation (like GW182 protein function in the repression of miRISCs) (Tritschler et al., 2010).

**FIGURE 1:**
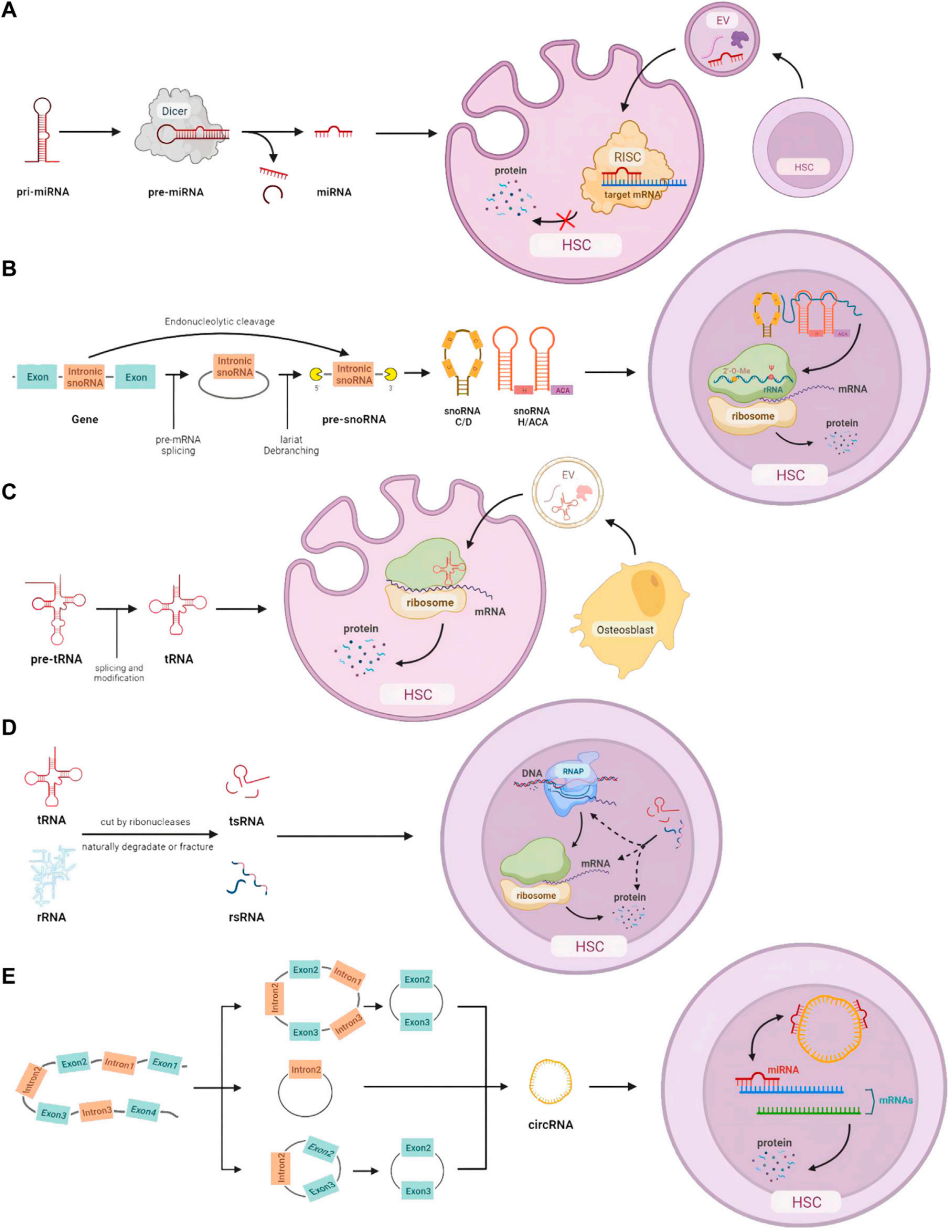
HSC homeostasis is regulated by small non-coding RNAs: **(A)** miRNA processing, intercellular transmission, and intracellular regulation of mRNA expression. **(B)** snoRNA processing and its function on rRNA modification. **(C)** tRNA processing, intercellular communication, and translation-related intracellular action. **(D)** The origin of tsRNA and rsRNA, and their potential control at the DNA, RNA, and protein levels. **(E)** Different cleavage formations of circRNA and its effects on miRNA.

According to a combination strategy using microarray analysis of miRNA expression and bioinformatic prediction of mRNA targets, different miRNAs fine-tune each stage of hematopoiesis. The inhibitory function is from the miR-128 and miR-181 in original hematopoietic lineages to miR-16, miR-103, and miR-107 in later progenitor cells proliferation (Georgantas et al., 2007). miRNAs are crucial in controlling the self-renewal and differentiation of stem cells, by inhibiting the translation of certain mRNAs in stem cells and differentiated daughter cells (Gangaraju and Lin, 2009). For example, ectopic expression of mir-150 can result in the reduction of mature B cells by downregulating MYB expression in hematopoietic stem cell progenitors (He et al., 2014). In addition, intercellular transportation of miRNAs has been found through the media of exosomes (EVs), playing a key role in immune evasion and cancer spread in solid tumors, as well as the pathological progression of many hematological malignancies (Van Niel et al., 2018); (Wu et al., 2017); (Caivano et al., 2017). Results from bioinformatics analysis and gain- and loss-of-function assays indicate that miR-548ac generated from EVs of AML cells suppresses hematopoiesis *via* TRIM28-dependent STAT3 activation (Zhao et al., 2022). To summarize, miRNAs are involved in the initiation and progression of blood diseases by intercellular delivery and regulation of related mRNA translation at different stages of hematopoiesis (Figure 1A).

### 2.2 snoRNAs

Small nucleolar RNAs (snoRNAs) with a common length of 60–300 nts are mainly in the nucleolus of eukaryotic cells. Based on the structure and their location, snoRNAs can be categorized into three subtypes: H/ACA box snoRNAs, C/D box snoRNAs, and scaRNAs (Huang et al., 2022). Most snoRNA genes are located in intronic regions and transcribed by RNA polymerase II. Some snoRNAs can also be derived from the intronic region of long non-coding RNA (lncRNA). After being detached from introns, pre-snoRNAs are further processed with exonuclease to remove excess sequences and form mature snoRNAs. The signal sequence inside snoRNAs directs snoRNAs to bind to corresponding proteins and form snoRNP complexes. In addition to the directing function in 2′-O-Methylation and pseudouridylation modification on rRNA separately, H/ACA box snoRNA and C/D box snoRNA are also involved in many physiological processes including RNA splicing, translation, oxidative stress response, etc. (Williams and Farzaneh, 2012) (Figure 1B).

In recent years, with the development of sequencing technology, more and more data show that snoRNAs are dysregulated in many diseases including hematological malignancies, solid tumors, hereditary diseases, and metabolic diseases (Williams and Farzaneh, 2012); (Liu et al., 2020a); (Verbeek et al., 2022). In a study of snoRNAs from 33 patients with acute myeloid leukemia, the probability of snoRNA dysregulation was not high, with only 9.3% of C/D box snoRNAs and 0.9% of H/ACA box snoRNAs found to be significantly increased or decreased (including some putative novel snoRNAs, using a rigorous 5% false discovery rate). However, some snoRNAs showed cell type-specific expression patterns among hematopoietic cell populations. For example, the C/D box snoRNA cluster in the imprinted Dlk1-Dio3 region is gradually lost during granulocytic differentiation (Warner et al., 2015). The expression of snoRNA is discovered to be regulated throughout hematopoiesis following the clue of homeostasis signals (Warner et al., 2018). For example, mutations in DDX41, an enzyme essential for snoRNA processing, ribosome assembly, and protein synthesis, are one of the most common causes of adult myelodysplastic syndrome. It was reported that knockout of DDX41 caused majority dysregulation of H/ACA box snoRNA processing and alterations in expression of series snoRNA, leading to defects in rRNA processing and ribosome translation (Chlon et al., 2021). A H/ACA box snoRNA, ACA11, was verified to overexpress in t (4; 14)-associated multiple myeloma, leading to tumorigenesis and drug resistance (Chu et al., 2012). Considering the recent development of therapeutics targeted at RNAs, snoRNAs exhibit great potential as pharmacological targets, either alone or in the context of immunotherapies (van der Werf et al., 2021).

### 2.3 tRNAs

The length of transfer RNAs (tRNAs) ranges from 76 to 90 nt, with the cloverleaf-like secondary structure and “L”-shaped tertiary structure (Suzuki, 2021). As an essential molecule in mRNA translation, tRNAs can identify the codon on mRNA by its anticodon and carry the corresponding amino acid to ribosomes to form a new polypeptide chain. tRNAs need to maintain considerable conservation and specific abundance according to the codon requirement.

Recent studies revealed that modifications of tRNAs that are located either on the anticodon loop or “elbow” part in the tertiary structure are vital. There is also evidence showing relationships between dysregulation of tRNA and various diseases such as cancer, neurological, and metabolic diseases (Santos et al., 2019). Recent research has identified tRNAs as an important regulator in gene expression during hematopoiesis (Lee et al., 2022). For instance, Elp3 is a gene coding acetyltransferase of tRNA, the lack of which results in the death of committing progenitors and further bone marrow failure (Rosu et al., 2021). tRNAs are also found to be directly packaged and transported through extracellular vesicles, which affects the functions of HSCs in the niche. Artificial synthesis of 5’ tiRNA with primary GMPs and Cy3-label was found predominantly rich in EVs produced by osteoblasts, stimulating protein translation, cell proliferation, and myeloid differentiation when transferred to granulocyte-monocyte progenitors (Kfoury et al., 2021). It is important for human bodies to regulate hematopoiesis and the immune system depending on tRNAs, which can control the mRNA translation and several extra- and intracellular molecule signaling pathways (Figure 1C).

### 2.4 tsRNAs and rsRNAs

It is the common feature of non-canonical sncRNAs that most of them are derived from other RNAs with longer length. Thanks to the development of novel technologies (LC-MS/MS, PANDORA-seq, etc.), the detectable level of modified sncRNAs experienced a dramatic increase (Shi et al., 2021). Recent research about sncRNAs focuses on transfer RNA-derived small RNA (tsRNA) and ribosomal RNA-derived small RNA (rsRNA), which have a potential earlier position in the evolution with widespread distributions in ancient unicellular organisms (Shi et al., 2022). Genetic conditions and environmental factors play an important role in their modification, which have been linked to many types of diseases, including cancer, immune system dysfunction, and neurological disorders (Figure 1D).

Evidence that the dysregulation of tsRNA biogenesis is involved in the pathogenesis of chronic lymphocytic leukemia has been found in primary B cell samples taken from patients with the disease as compared to normal B cells obtained from healthy human donors (Veneziano et al., 2019). Studies have also demonstrated that tsRNA-mediated translation is important for the regulation of hematopoietic stem cell commitment. PUS7 inactivation leads to aberrant tsRNA regulation, which functions to increase the biosynthesis of protein and further affects germ layer specification in embryonic stem cells (Guzzi et al., 2018). It is noticeable that locating sncRNAs in subcellular spaces remains a major challenge (Shi et al., 2022) because abundant RNA structures and modifications can hinder effective *in situ* hybridization. Designing probes with higher sensitivity and specificity would greatly assist this field.

### 2.5 circRNAs

Circular RNAs (circRNAs) were firstly discovered under the electron microscope in 1979 (Hsu and Coca-Prados, 1979). CircRNAs are a subtype of non-coding RNAs, which stably exist in a loop shape and lack free 3′and 5′ends. There are four types of circRNAs based on their origins: exon-derived circRNAs (ecRNAs), intron-derived circRNAs (ciRNAs), circRNAs with exons and introns (EIciRNAs), and intergenic circRNAs. CircRNA is synthesized depending on unconventional “reverse splicing.” Most circRNAs are made up of 1–5 exons of the corresponding mRNA coding region that has been “reverse spliced” at the 3′and 5′ends to create a continuous covalent ring structure (Salzman et al., 2012). Additionally, long non-coding RNAs (lncRNAs), reverse transcripts, intergenic regions, and introns can all be sources of circRNAs (Jeck et al., 2013). The miRNA sponge activity is the most well-known function of circRNAs. Some circRNAs contain numerous miRNA-binding sites in their nucleotide sequence, which can prevent miRNAs from binding to their conventional mRNA targets (Kristensen et al., 2019). Some circRNAs can bind with proteins as protein sponges (Ashwal-Fluss et al., 2014). It is noticeable that circRNAs in the nucleus usually participate in transcription regulation, splicing, and chromatin looping (Li et al., 2015); (Conn et al., 2017); (Liu et al., 2020b).

The type and number of circRNAs alter during the maturation of HSCs (Nicolet et al., 2018) (Figure 1E). Research has illustrated linkages between circRNA and cell proliferation in hematological malignancies. Based on the microassay, circMYBL2, one subtype of circRNA, showed a higher expression level in FLT3-ITD AML^+^. Knockdown of circMYBL2 effectively inhibited the proliferation of FLT3-ITD AML cells and overcame the acquired resistance to quizartinib^+^. It has been demonstrated that circMYBL2 controls FLT3 translation by recruiting PTBP1 to promote the progression of FLT3-internal tandem duplication acute myeloid leukemia (Sun et al., 2019).

The functions and pathways of these small non-coding RNAs in HSC are shown in Figure 1.

## 3 RNA modifications on small non-coding RNAs and their roles in hematopoiesis

Epitranscriptomics is a new field in recent research. Because of technical limitations, the modification mechanism and effect of small non-coding RNAs still need further exploration. Here, we list several classical modification types and illustrate their function and roles in hematopoiesis.

### 3.1 m6A

N6-methyladenosine (m6A) is the most common and conserved internal modification in eukaryotic RNAs. The writer complex of m6A methylation reaction, m6A methyltransferases, includes METTL3/14/16, RBM15/15B, ZC3H3, VIRMA, CBLL1, WTAP, and KIAA1429. There are two kinds of demethylases, or say erasers, fat mass and obesity-associated protein (FTO) and alpha-ketoglutarate-dependent hydroxylases homolog 5 (ALKBH5) that rely on Fe^2+^ to remove the m6A. The readers include the YT521-B homology (YTH) domain family, IGF2BP1/2/3, and HNRNPA2B1. M6A functions by regulating translation and stability of mRNA.

**FIGURE 2:**
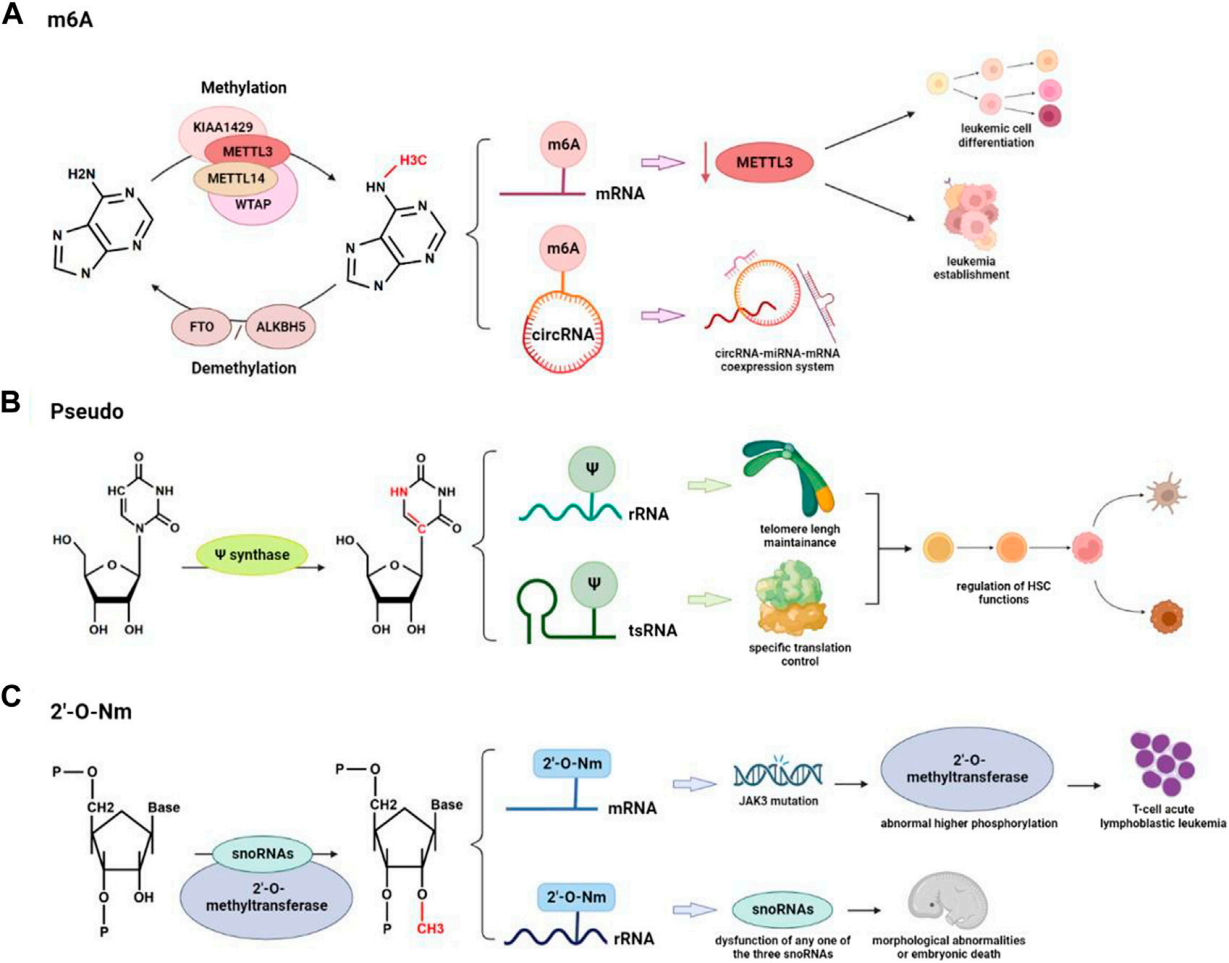
RNA modifications are involved in the regulation of hematopoiesis and hematological malignancies: **(A)** Reversible conversion of m6A modification and differential regulation function on mRNA and circRNA in hematologic cancers. **(B)** Pseudo is involved in the regulation of HSC by acting on rRNA to maintain telomere length or on tsRNA to modulate specific translation processes. **(C)** Abnormal 2′-O-Nm can cause mutations in other important genes and can also lead to severe morphological abnormalities and embryonic death.

The regulatory functions of m6A modification on mRNA level have been commonly identified in a generally negative correlation during the development of hematopoiesis and leukemogenesis (Yao et al., 2021) (Figure 2A). Research on immunodeficient mice revealed a specific pathway of METTL3 writer complex. The downregulation of METTL3 hindered the maintenance of leukemia, including cell cycle arrest, leukemic cell differentiation, and failure to establish leukemia (Barbieri et al., 2017). YTHDC1, a nuclear reader of m6A, was reported to be highly expressed in the samples from AML patients, which links to the cell cycle and proliferation of the malignant clone (Sheng et al., 2021). Based on super-low input m6A-seq (SLIM-seq), Zhang and his colleagues discovered IGF2BP2, another crucial m6A reader that is abundantly expressed in long-term hematopoietic stem cells and keeps the stability of downstream m6A-modified mRNA (One important downstream target Bmi1, for example, regulates mitochondria-related genes to maintain hematopoietic stem cell function) (Yin et al., 2022). M6A is also detected on small non-coding RNAs (circRNA, miRNA, snoRNA, snRNA, and tRNA). Hsa_circ_0004277 has been detected to be significantly downregulated in patients with acute myeloid leukemia (Li et al., 2017). Research also obtained the circRNA expression profile with m6A modification and predicted the network of circRNA-miRNA-mRNA coexpression system. FoxO and ErbB are two potential pathways according to KEGG pathway analysis (Issah et al., 2022). Based on scDART-seq, m6A was also reported as a potential driving force for the co-regulation of subpopulations of cells, which successfully distinguished groups of cells according to the m6A pattern (Tegowski et al., 2022). However, the specific regulatory mechanism and the functions that m6A and miRNA have in hematopoiesis and leukemia development are still worth further investigation. According to current research, pharmacological interventions targeting m6A regulators to correct dysregulation may offer new therapeutic opportunities for anticancer treatments (Gao et al., 2021). The first small compounds that were found to target m6A regulators are FTO inhibitors. One novel FTO inhibitor, FB23-2, was designed to bind with FTO and showed a significant suppressive effect on the proliferation and enhancement of human AML cell line apoptosis *in vitro* (Huang et al., 2019).

### 3.2 Pseudouridylation

Pseudouridylation is the most common post-transcriptional RNA modification where a uridine residue is isomerized into a pseudouridine (Ψ) through pseudouridine synthase. The distribution of pseudouridines covers tRNA, rRNA, snRNA, snoRNA, and mRNA. There are three characteristics of *Ψ* that differ it from uridine and other nucleotides: 1. more rigid phosphodiester backbone of RNA; 2. more stable Ψ-A base pairs through certain effects on base stacking and water coordination; 3. Better thermal stability. Ψ‐A, Ψ‐G, Ψ‐U, and Ψ‐C pairs are formed at several sites in structured RNAs that are conserved across species to stabilize important RNA duplexes and improve RNA-protein interactions (Lin et al., 2021).

Mutations in DKC1, coding gene for pseudouridine synthase, are reported to reduce pseudouridylation and processing of rRNA in *Drosophila*, resulting in Hoyeraal-Hreidarsson syndrome with the symptoms of immunodeficiency, growth retardation, and microcephaly (Ruggero et al., 2003). Accompanying telomere dysfunction, bone marrow failure is one symptom of Hoyeraal-Hreidarsson syndrome (Fok et al., 2017). It has been reported that pseudouridylation on rRNAs has effects on hematopoiesis through the regulation of telomere shortening. Recent studies also indicated that *Ψ* synthase PUS7 controls cell proliferation and protein synthesis in stem cells, which can also regulate the biogenesis of tsRNAs. Furthermore, Guzzi et al. (2018) reported that PUS7-mediated *Ψ* can activate particular tsRNAs (mTOGs) to inhibit translation in stem cells. Loss of PUS7 and mTOGs affects hematopoietic commitment and early embryogenesis *in vitro*. Therefore, pseudouridine on tsRNA can regulate hematopoiesis through control of specific mRNA translation in stem cells (HSPCs) (Dimitrova et al., 2019) (Figure 2B). Pseudouridylation has already been applied in the production of mRNA vaccines due to its stability feature (Morais et al., 2021). High concentrations of *Ψ* have also been found in the urine, plasma, and salivary metabolites of patients with ovarian, prostate, and colon cancer, indicating that this substance may be useful as a biomarker in liquid non-invasive biopsies for the early detection of cancer (Krstulja et al., 2017); (Sridharan et al., 2019); (Zeleznik et al., 2020).

### 3.3 2’-O-methylation (Nm)

2′-O-methylation (Nm) is the modification of adding a methyl group (-CH3) to the 2’ hydroxyl (-OH) of the ribose moiety, which can occur either during or after the transcription. Nm has been found in many types of RNAs (rRNA, tRNA, snRNA, mRNA, etc.), and can affect RNA molecules on their structure, stability, and interactions, thus regulating a range of cellular processes, such as self vs non-self-recognition and epigenetic gene regulation (Dimitrova et al., 2019).

It is already known that small nucleolar RNAs (snoRNAs) can direct internal Nm modification to further control mRNA and protein expression. It is reported that two box C/D of snoRNAs and the 2’-O-methyltransferase fibrillarin act together and make Nm modification in the protein-coding region of peroxidasin (Pxdn) (Elliott et al., 2019). The disease of T-cell acute lymphoblastic leukemia can be caused by mutations in the Janus kinase 3 (JAK3) gene, activity inhibition of which is found to link with abnormal higher phosphorylation level of CMTR1, another type of 2’-O-methyltransferase (Dimitrova et al., 2019).

Similar processes also happen during the Nm modification of rRNA. Research on the zebrafish identified the importance of rRNA Nm modifications for vertebrate development. Dysfunction on any one of the three snoRNAs: SNORD26, SNORD44, or SNORD78 results in severe morphological abnormalities and embryonic death (Higa-Nakamine et al., 2012). Through a thorough analysis of the rRNA Nm landscape from 94 patients with AML, recent studies also confirmed the plasticity of rRNA Nm that shifts protein translation toward a leukemia stem cell phenotype (Zhou et al., 2023) (Figure 2C). It is interesting to note that in a population of ribosomes, the amounts of modification at the various 2-O methylatable sites are not necessarily the same, but the center of ribosomal subunits frequently contains fully ribomethylated regions. These constitutive locations have relative conservations across a range of cell types and conditions, indicating that ribomethylation at these positions is essential for ribosome biogenesis and function (Barros-Silva et al., 2021).

## 4 Cutting-edge technologies for detection of small RNA and RNA modifications

The breakthroughs of research on small RNA modification and related diseases cannot be separated from the development of sequencing technology. Before the introduction of high-throughput RNA sequencing, Northern Blot was used to assess the level of RNA expression (Hayes et al., 1989). The quantity of RNA can be measured by reverse transcribed polymerase chain reaction (Nolan et al., 2006). Primer extension design and Poly A sizing assay, respectively, were widely employed to detect the 5′and 3′ends of mRNA (Wilkinson et al., 2006); (Mercer and Wake, 1985). Microarray analysis was used to evaluate the level of mRNA expression and modification (Hughes et al., 2006). Isotope labeling in metabolomics can also be used in RNA modification studies (Asadi-Atoi et al., 2019). As for the detection of RNA-protein interaction, it was commonly based on the technology of yeast three-hybrid system or RNA footprinting and modification interference analysis (König et al., 2007); (Clarke, 1999).

Since its application 10 years ago, next-generation RNA sequencing (RNA-seq) technologies provide accurate methodologies to obtain information of the whole transcriptomes, including mRNA, small RNA (sRNA), and long non-coding RNA (lncRNA). Small RNA sequencing based on NGS technologies helps scientists to isolate and collect information about non-coding RNA molecules, which is powerful to profile and discover new types of small RNAs (Ozsolak and Milos, 2011).

However, because of the RNA modifications that interfere with adapter ligation and reverse transcription processes, there are difficulties in the detection and sequence of non-coding small RNA. As an advanced sequencing and analysis technology, PANDORA-seq has developed enzymatic treatment methods to handle certain RNA modifications. For instance, the demethylating AlkB and its mutant variants are employed to assure reverse transcription. In order to facilitate adapter ligation for RNA-seq of small and large RNAs, T4 polynucleotide kinase (T4PNK) is applied. It is also noticeable that T4PNK pretreatment of RNA should be applied according to the specific requirement (T4PNK is not recommended for research on miRNA due to the higher level of non-miRNA reads) (Shigematsu and Kirino, 2022). Based on PANDORA-seq, tsRNA and rsRNA have been observed largely expressed in the mouse brain, liver, and HeLa cells (Liu et al., 2023).

Various specialized techniques have been developed and refined for detecting each RNA modification type. RibOxi-seq, for instance, is effective in characterizing rRNA Nm sites with minimal input RNA material and sequencing depth while maintaining good accuracy. The production of RNA fragments with random 3′-ends, accompanied by periodate oxidation of all molecules terminating in 2′, 3′-OH groups, is crucial for this approach. As a result, only RNAs with 2′-OMe groups at their 3′ends can be sequenced in RibOxi-seq (Zhu et al., 2017). Relevant work also applied the machine learning algorithm (Random Forest) to develop a predictive model for analysis of RNA’s 2′-O-methylated sites based on RiboMethSeq datasets (Pichot et al., 2022). For the detection of m6A modification, Meyer and his group successfully developed and optimized scDART-seq, achieving the goal of single-cell m6A sequencing (Tegowski and Meyer, 2022). This technology applied APOBEC-YTH fusion protein to identify the m6A site in a non-antibody-dependent method. A synthetic modification-free RNA library was also developed as a negative control that comprehensively evaluated the existing RNA modification detection methods and provided a systematic solution for the accurate identification of modification sites (Zhang et al., 2021). To avoid the bias and limitations caused by these approaches, nanopore RNA sequencing was optimized to discriminate and identify different RNA modifications in native RNA, including m6A, pseudouridine and Nm, etc. In a creative project, by detecting single-molecule reads with varied current intensity and trace profiles, researchers estimate per-site alteration stoichiometries based on the analysis of distinctive base-calling “error” signals in the nanopore data (Begik et al., 2021).

Previous studies usually used specific cell surface markers to distinguish different subtypes of progenitor populations. Limitations of buck cell sequencing have been overcome by single-cell sequencing (scRNA-seq), which can identify gene expression patterns in uniform cell populations (Grün and Van Oudenaarden, 2015). Based on scRNA-seq, scientists discovered the cellular heterogeneity among populations and traced cell fate to reconstruct developmental trajectories. Furthermore, scRNA also solved the problem of collecting human hematopoietic cells with inadequate sample sizes, enabling investigations that were previously carried out in mouse models to be replicated in human cells. Single-cell techniques have also been applied to profile small non-coding RNA expressions. For example, Faridani et al. (2016) reported single-cell sequencing method to detect small-RNA transcriptome. To counteract PCR randomness and count RNA molecules, this technique made use of a library created *via* RNA species linkage and a unique molecular identifier. The author used this method to research small-RNA expression in naive and primed human embryonic stem cells, as well as cancer cells (Faridani et al., 2016).

Small RNAs are important components used as guide RNA (gRNA) in CRISPR/Cas9 systems. After transducing gRNA and Cas proteins into cells, the Cas9 protein binds to gRNA and attaches the target DNA sequence to perform DNA double-strand cleavage. When a break occurs in the DNA sequence, it activates the DNA repair mechanisms and results in mutations. CRISPR/Cas9-based gene screening, a large-scale unbiased genetic loss-of-function screening method designed to unbiased screen functional genes, was also applied in investigating the epigenetic regulation in hematopoiesis and leukemia (Yamauchi et al., 2018). With various modifications on the dCas9/sgRNA complex, CRISPR interference (CRISPRi), CRISPR activation (CRISPRa), and CRISPRon/off techniques were developed successively, while the first 2 were widely used for screening of non-coding RNAs. To investigate functions and related pathways about modifications on small non-coding RNAs, technologies such as CRISPR/Cas9 and RNA interference can edit or silence upstream genes that regulate the maturity and modification of small non-coding RNA to study relative functions and pathways. Based on RNAi, METTL3 was found to be responsible for m6A modification in the zebrafish embryos. In the experiment of Zhang et al. (2017), there was a blocked presence of HSPC when the m6A was significantly decreased. All these technologies are powerful tools to help with future studies (Table 1).

**TABLE 1:** Cutting-edge technologies applied to study sncRNAs and RNA modifications.

Technology	Application	Ref
*Small RNA sequencing*	Analyze and discover new types of small RNAs	Ozsolak and Milos (2011)
*PANDORA-seq*	Detect and sequence non-coding small RNAs	Shigematsu and Kirino (2022)
*RibOxi-seq, scDART-seq*	Detect and sequence modifications	Zhu et al. (2017), Tegowski and Meyer (2022)
*Modification-free RNA library*	Act as negative control and improve accuracy of modification identifications	Zhang et al. (2021)
*Nanopore RNA sequencing*	Distinguish and recognize different RNA modifications	Begik et al. (2021)
*Machine learning algorithm*	Analyze 2′-O-methylated sites in RNA based on datasets	Pichot et al. (2022)
*Single-cell sequencing*	A. identify gene expression patterns in cell populations; B. enable detection with small sample size; C. profile small non-coding RNA expressions	Grün and Van Oudenaarden (2015), Faridani et al. (2016)
*CRISPR/Cas9*	A. Gene editing; B. functional gene screen; C. non-coding RNA screen	Yamauchi et al. (2018), Zhang et al. (2017)

## 5 Conclusion and future perspectives

In this study, we have formulated the basic concept of HSCs and small non-coding RNA modifications. We also listed recent research results and summarized typical modifications (m6A, Pseudo, and Nm) found on small non-coding RNAs associated with HSCs-related diseases. Many studies have indicated the important function of small non-coding RNAs that regulates gene expression to maintain homeostasis. Modifications of the small non-coding RNAs are still under research. The research area about their indirect effects on gene expression through the regulation of small non-coding RNAs remains largely unknown.

Nowadays, many diseases like cancer, mitochondrial diseases, and neurological disorders have been detected to be associated with small non-coding RNA modifications. These characteristics make small non-coding RNA modifications potential diagnostic and prognostic biomarkers. For example, hypomodified tRNA was found in colorectal cancer, which induces ribosome frameshifting and further causes TYW2 epigenetic inactivation through non-sense-mediated RNA decay (Tavares et al., 2021). Research on modifications of small non-coding RNA can also be helpful for disease treatment in the future. Medicine that targets molecular pathways with dysregulated specific small non-coding RNA can alleviate symptoms and control disease development.

## References

[B1] Asadi-Atoi P. Barraud P. Tisne C. Kellner S. (2019). Benefits of stable isotope labeling in RNA analysis. Biol. Chem. 400, 847–865. 10.1515/hsz-2018-0447 30893050

[B2] Ashwal-Fluss R. Meyer M. Pamudurti N. R. Ivanov A. Bartok O. Hanan M. (2014). circRNA biogenesis competes with pre-mRNA splicing. Mol. Cell 56, 55–66. 10.1016/j.molcel.2014.08.019 25242144

[B3] Barbieri I. Tzelepis K. Pandolfini L. Shi J. Millan-Zambrano G. Robson S. C. (2017). Promoter-bound METTL3 maintains myeloid leukaemia by m6A-dependent translation control. Nature 552, 126–131. 10.1038/nature24678 29186125 PMC6217924

[B4] Barros-Silva D. Klavert J. Jenster G. Jeronimo C. Lafontaine D. L. J. Martens-Uzunova E. S. (2021). The role of OncoSnoRNAs and ribosomal RNA 2’-O-methylation in cancer. RNA Biol. 18, 61–74. 10.1080/15476286.2021.1991167 34775914 PMC8677010

[B5] Begik O. Lucas M. C. Pryszcz L. P. Ramirez J. M. Medina R. Milenkovic I. (2021). Quantitative profiling of pseudouridylation dynamics in native RNAs with nanopore sequencing. Nat. Biotechnol. 39, 1278–1291. 10.1038/s41587-021-00915-6 33986546

[B6] Caivano A. La Rocca F. Laurenzana I. Trino S. De Luca L. Lamorte D. (2017). Extracellular vesicles in hematological malignancies: From biology to therapy. Int. J. Mol. Sci. 18, 1183. 10.3390/ijms18061183 28574430 PMC5486006

[B7] Carrelha J. Meng Y. Kettyle L. M. Luis T. C. Norfo R. Alcolea V. (2018). Hierarchically related lineage-restricted fates of multipotent haematopoietic stem cells. Nature 554, 106–111. 10.1038/nature25455 29298288

[B8] Carvalho T. L. Mota-Santos T. Cumano A. Demengeot J. Vieira P. (2001). Arrested B lymphopoiesis and persistence of activated B cells in adult interleukin 7(-/)- mice. J. Exp. Med. 194, 1141–1150. 10.1084/jem.194.8.1141 11602642 PMC2193519

[B9] Chlon T. M. Stepanchick E. Hershberger C. E. Daniels N. J. Hueneman K. M. Kuenzi Davis A. (2021). Germline DDX41 mutations cause ineffective hematopoiesis and myelodysplasia. Cell Stem Cell 28, 1966–1981.e6. 10.1016/j.stem.2021.08.004 34473945 PMC8571055

[B10] Chu L. Su M. Y. Maggi L. B. Jr Lu L. Mullins C. Crosby S. (2012). Multiple myeloma-associated chromosomal translocation activates orphan snoRNA ACA11 to suppress oxidative stress. J. Clin. Invest. 122, 2793–2806. 10.1172/JCI63051 22751105 PMC3408744

[B11] Chung S. S. Park C. Y. (2017) Aging, hematopoiesis, and the myelodysplastic syndromes. Hematol. Am. Soc. Hematol. Educ. Progr. 2017, 73–78. 10.1182/asheducation-2017.1.73 PMC6142578 29222239

[B12] Clarke P. A. (1999). RNA footprinting and modification interference analysis. Methods Mol. Biol. 118, 73–91. 10.1385/1-59259-676-2:73 10549516

[B13] Conn V. M. Hugouvieux V. Nayak A. Conos S. A. Capovilla G. Cildir G. (2017). A circRNA from SEPALLATA3 regulates splicing of its cognate mRNA through R-loop formation. Nat. plants 3, 17053. 10.1038/nplants.2017.53 28418376

[B14] Dimitrova D. G. Teysset L. Carré C. (2019). RNA 2’-O-methylation (Nm) modification in human diseases. Genes (Basel). 10, 117. 10.3390/genes10020117 30764532 PMC6409641

[B15] Elliott B. A. Ho H. T. Ranganathan S. V. Vangaveti S. Ilkayeva O. Abou Assi H. (2019). Modification of messenger RNA by 2′-O-methylation regulates gene expression *in vivo* . Nat. Commun. 10, 3401–3409. 10.1038/s41467-019-11375-7 31363086 PMC6667457

[B16] Fabian M. R. Sonenberg N. (2012), The mechanics of miRNA-mediated gene silencing: A look under the hood of miRISC. Nat. Struct. Mol. Biol. 19, 586–593. 10.1038/nsmb.2296 22664986

[B17] Faridani O. R. Abdullayev I. Hagemann-Jensen M. Schell J. P. Lanner F. Sandberg R. (2016). Single-cell sequencing of the small-RNA transcriptome. Nat. Biotechnol. 34, 1264–1266. 10.1038/nbt.3701 27798564

[B18] Fok W. C. Niero E. L. d. O. Dege C. Brenner K. A. Sturgeon C. M. Batista L. F. Z. (2017). p53 mediates failure of human definitive hematopoiesis in dyskeratosis congenita. Stem Cell Rep. 9, 409–418. 10.1016/j.stemcr.2017.06.015 PMC5550027 28757166

[B19] Gangaraju V. K. Lin H. (2009). MicroRNAs: Key regulators of stem cells. Nat. Rev. Mol. Cell Biol. 10, 116–125. 10.1038/nrm2621 19165214 PMC4118578

[B20] Gao R. Ye M. Liu B. Wei M. Ma D. Dong K. (2021). m6A modification: A double-edged sword in tumor development. Front. Oncol. 11, 679367–679412. 10.3389/fonc.2021.679367 34381710 PMC8350482

[B21] Georgantas R. W. Hildreth R. Morisot S. Alder J. Liu C. g. Heimfeld S. (2007). CD34+ hematopoietic stem-progenitor cell microRNA expression and function: A circuit diagram of differentiation control. Proc. Natl. Acad. Sci. U. S. A. 104, 2750–2755. 10.1073/pnas.0610983104 17293455 PMC1796783

[B22] Grün D. Van Oudenaarden A. (2015). Design and analysis of single-cell sequencing experiments. Cell 163, 799–810. 10.1016/j.cell.2015.10.039 26544934

[B23] Guzzi N. Cieśla M. Ngoc P. C. T. Lang S. Arora S. Dimitriou M. (2018). Pseudouridylation of tRNA-derived fragments steers translational control in stem cells. Cell 173, 1204–1216.e26. 10.1016/j.cell.2018.03.008 29628141

[B24] Hayes P. C. Wolf C. R. Hayes J. D. (1989). Blotting techniques for the study of DNA, RNA, and proteins. BMJ 299, 965–968. 10.1136/bmj.299.6705.965 2478239 PMC1837801

[B25] He Y. Jiang X. Chen J. (2014). The role of miR-150 in normal and malignant hematopoiesis. Oncogene 33, 3887–3893. 10.1038/onc.2013.346 23955084

[B26] Higa-Nakamine S. Suzuki T. Uechi T. Chakraborty A. Nakajima Y. Nakamura M. (2012). Loss of ribosomal RNA modification causes developmental defects in zebrafish. Nucleic Acids Res. 40, 391–398. 10.1093/nar/gkr700 21908402 PMC3245925

[B27] Ho T. T. Warr M. R. Adelman E. R. Lansinger O. M. Flach J. Verovskaya E. V. (2017). Autophagy maintains the metabolism and function of young and old stem cells. Nature 543, 205–210. 10.1038/nature21388 28241143 PMC5344718

[B28] Hsu M. T. Coca-Prados M. (1979). Electron microscopic evidence for the circular form of RNA in the cytoplasm of eukaryotic cells. Nature 280, 339–340. 10.1038/280339a0 460409

[B29] Huang Y. Su R. Sheng Y. Dong L. Dong Z. Xu H. (2019). Small-molecule targeting of oncogenic FTO demethylase in acute myeloid leukemia. Cancer Cell 35, 677–691.e10. 10.1016/j.ccell.2019.03.006 30991027 PMC6812656

[B30] Huang Z. Du Y. Wen J. Lu B. Zhao Y. (2022). snoRNAs: functions and mechanisms in biological processes, and roles in tumor pathophysiology. Cell death Discov. 8, 259. 10.1038/s41420-022-01056-8 35552378 PMC9098889

[B31] Hughes T. R. Hiley S. L. Saltzman A. L. Babak T. Blencowe B. J. (2006). Microarray analysis of RNA processing and modification. Methods Enzymol. 410, 300–316. 10.1016/S0076-6879(06)10014-2 16938557

[B32] Issah M. A. Wu D. Zhang F. Zheng W. Liu Y. Chen R. (2022). Expression profiling of N6-methyladenosine modified circRNAs in acute myeloid leukemia. Biochem. Biophys. Res. Commun. 601, 137–145. 10.1016/j.bbrc.2022.02.087 35247767

[B33] Jeck W. R. Sorrentino J. A. Wang K. Slevin M. K. Burd C. E. Liu J. (2013). Circular RNAs are abundant, conserved, and associated with ALU repeats. RNA 19, 141–157. 10.1261/rna.035667.112 23249747 PMC3543092

[B34] Kfoury Y. S. Ji F. Mazzola M. Sykes D. B. Scherer A. K. Anselmo A. (2021). tiRNA signaling via stress-regulated vesicle transfer in the hematopoietic niche. Cell Stem Cell 28, 2090–2103.e9. 10.1016/j.stem.2021.08.014 34551362 PMC8642285

[B35] König J. Julius C. Baumann S. Homann M. Goringer H. U. Feldbrugge M. (2007). Combining SELEX and the yeast three-hybrid system for *in vivo* selection and classification of RNA aptamers. RNA 13, 614–622. 10.1261/rna.334307 17283213 PMC1831868

[B36] Kristensen L. S. Andersen M. S. Stagsted L. V. W. Ebbesen K. K. Hansen T. B. Kjems J. (2019). The biogenesis, biology and characterization of circular RNAs. Nat. Rev. Genet. 20, 675–691. 10.1038/s41576-019-0158-7 31395983

[B37] Krol J. Loedige I. Filipowicz W. (2010). The widespread regulation of microRNA biogenesis, function and decay. Nat. Rev. Genet. 11, 597–610. 10.1038/nrg2843 20661255

[B38] Krstulja A. Lettieri S. Hall A. J. Roy V. Favetta P. Agrofoglio L. A. (2017). Tailor-made molecularly imprinted polymer for selective recognition of the urinary tumor marker pseudouridine. Macromol. Biosci. 17, 1700250. 10.1002/mabi.201700250 29144579

[B39] Lee A. K. Aifantis I. Thandapani P. (2022). Emerging roles for tRNAs in hematopoiesis and hematological malignancies. Trends Immunol. 43, 466–477. 10.1016/j.it.2022.03.009 35490133

[B40] Li D. Xue W. Li M. Dong M. Wang J. Wang X. (2018). VCAM-1 + macrophages guide the homing of HSPCs to a vascular niche. Nature 564, 119–124. 10.1038/s41586-018-0709-7 30455424 PMC6492262

[B41] Li W. Zhong C. Jiao J. Li P. Cui B. Ji C. (2017). Characterization of hsa_circ_0004277 as a new biomarker for acute myeloid leukemia via circular RNA profile and bioinformatics analysis. Int. J. Mol. Sci., Vol. 18, 597 10.3390/ijms18030597 28282919 PMC5372613

[B42] Li Z. Huang C. Bao C. Chen L. Lin M. Wang X. (2015). Exon-intron circular RNAs regulate transcription in the nucleus. Nat. Struct. Mol. Biol. 22, 256–264. 10.1038/nsmb.2959 25664725

[B43] Lin T. Y. Mehta R. Glatt S. (2021). Pseudouridines in RNAs: Switching atoms means shifting paradigms. FEBS Lett. 595, 2310–2322. 10.1002/1873-3468.14188 34468991 PMC9290505

[B44] Liu J. Shi J. Hernandez R. Li X. Konchadi P. Miyake Y. (2023). Paternal phthalate exposure-elicited offspring metabolic disorders are associated with altered sperm small RNAs in mice. Environ. Int. 172, 107769. 10.1016/j.envint.2023.107769 36709676 PMC10194346

[B45] Liu Y. Ruan H. Li S. Ye Y. Hong W. Gong J. (2020). The genetic and pharmacogenomic landscape of snoRNAs in human cancer. Mol. Cancer 19, 108. 10.1186/s12943-020-01228-z 32576192 PMC7313177

[B46] Liu Y. Su H. Zhang J. Feng C. Han F. (2020). Back-spliced RNA from retrotransposon binds to centromere and regulates centromeric chromatin loops in maize. PLoS Biol. 18, e3000582. 10.1371/journal.pbio.3000582 31995554 PMC7010299

[B47] Mattick J. S. (2001). Non-coding RNAs: The architects of eukaryotic complexity. EMBO Rep. 2, 986–991. 10.1093/embo-reports/kve230 11713189 PMC1084129

[B48] Mejia-Ramirez E. Florian M. C. (2020). Understanding intrinsic hematopoietic stem cell aging. Haematologica 105, 22–37. 10.3324/haematol.2018.211342 31806687 PMC6939535

[B49] Mercer J. F. B. Wake S. A. (1985). An analysis of the rate of metallothionein mRNA poly(A)-shortening using RNA blot hybridization. Nucleic Acids Res. 13, 7929–7943. 10.1093/nar/13.22.7929 2866488 PMC322101

[B50] Morais P. Adachi H. Yu Y. T. (2021). The critical contribution of pseudouridine to mRNA COVID-19 vaccines. Front. Cell Dev. Biol. 9, 789427. 10.3389/fcell.2021.789427 34805188 PMC8600071

[B51] Nicolet B. P. Engels S. Aglialoro F. van den Akker E. von Lindern M. Wolkers M. C. (2018). Circular RNA expression in human hematopoietic cells is widespread and cell-type specific. Nucleic Acids Res. 46, 8168–8180. 10.1093/nar/gky721 30124921 PMC6144802

[B52] Nolan T. Hands R. E. Bustin S. A. (2006). Quantification of mRNA using real-time RT-PCR. Nat. Protoc. 1, 1559–1582. 10.1038/nprot.2006.236 17406449

[B53] Notta F. Zandi S. Takayama N. Dobson S. Gan O. I. Wilson G. (2016). Distinct routes of lineage development reshape the human blood hierarchy across ontogeny. Science 80-, aab2116. 10.1126/science.aab2116 PMC4816201 26541609

[B54] Ozsolak F. Milos P. M. (2011). RNA sequencing: Advances, challenges and opportunities. Nat. Rev. Genet. 12, 87–98. 10.1038/nrg2934 21191423 PMC3031867

[B55] Pichot F. Marchand V. Helm M. Motorin Y. (2022). Machine learning algorithm for precise prediction of 2’-O-methylation (Nm) sites from experimental RiboMethSeq datasets. Methods 203, 311–321. 10.1016/j.ymeth.2022.03.007 35314341

[B56] Pinho S. Frenette P. S. (2019). Haematopoietic stem cell activity and interactions with the niche. Nat. Rev. Mol. Cell Biol. 20, 303–320. 10.1038/s41580-019-0103-9 30745579 PMC6483843

[B57] Prashad S. L. Calvanese V. Yao C. Y. Kaiser J. Wang Y. Sasidharan R. (2015). GPI-80 defines self-renewal ability in hematopoietic stem cells during human development. Cell Stem Cell 16, 80–87. 10.1016/j.stem.2014.10.020 25465114 PMC4520393

[B58] Rosu A. El Hachem N. Rapino F. Rouault-Pierre K. Jorssen J. Somja J. (2021). Loss of tRNA-modifying enzyme Elp3 activates a p53-dependent antitumor checkpoint in hematopoiesis. J. Exp. Med. 218, e20200662. 10.1084/jem.20200662 33507234 PMC7849823

[B59] Ruggero D. Grisendi S. Piazza F. Rego E. Mari F. Rao P. H. (2003). Dyskeratosis congenita and cancer in mice deficient in ribosomal RNA modification. Sci. (80-. ) 299, 259–262. 10.1126/science.1079447 12522253

[B60] Salzman J. Gawad C. Wang P. L. Lacayo N. Brown P. O. (2012). Circular RNAs are the predominant transcript isoform from hundreds of human genes in diverse cell types. PLoS One 7, e30733. 10.1371/journal.pone.0030733 22319583 PMC3270023

[B61] Santos M. Fidalgo A. Varanda A. S. Oliveira C. Santos M. A. S. (2019). tRNA deregulation and its consequences in cancer. Trends Mol. Med. 25, 853–865. 10.1016/j.molmed.2019.05.011 31248782

[B62] Sheng Y. Wei J. Yu F. Xu H. Yu C. Wu Q. (2021). A critical role of nuclear m6A reader YTHDC1 in leukemogenesis by regulating MCM complex-mediated DNA replication. Blood 138, 2838–2852. 10.1182/blood.2021011707 34255814 PMC8718631

[B63] Shi J. Zhang Y. Tan D. Zhang X. Yan M. Zhang Y. (2021). PANDORA-seq expands the repertoire of regulatory small RNAs by overcoming RNA modifications. Nat. Cell Biol. 23, 424–436. 10.1038/s41556-021-00652-7 33820973 PMC8236090

[B64] Shi J. Zhou T. Chen Q. (2022). Exploring the expanding universe of small RNAs. Nat. Cell Biol. 24, 415–423. 10.1038/s41556-022-00880-5 35414016 PMC9035129

[B65] Shigematsu M. Kirino Y. (2022). Making invisible RNA visible: Discriminative sequencing methods for RNA molecules with specific terminal formations. Biomolecules 12, 611. 10.3390/biom12050611 35625540 PMC9138997

[B66] Slukvin I. D’Souza S. S. Kumar A. (2022). Induced pluripotent stem cells–derived hematopoietic progenitors for cellular immunotherapies. iPSC Deriv. Progenitors, 233–263. 10.1016/B978-0-323-85545-7.00007-7

[B67] Sridharan G. Ramani P. Patankar S. Vijayaraghavan R. (2019). Evaluation of salivary metabolomics in oral leukoplakia and oral squamous cell carcinoma. J. Oral Pathol. Med. 48, 299–306. 10.1111/jop.12835 30714209

[B68] Sun W. Julie Li Y. S. Huang H. Da Shyy J. Y. J. Chien S. (2010). microRNA: a master regulator of cellular processes for bioengineering systems. Annu. Rev. Biomed. Eng. 12, 1–27. 10.1146/annurev-bioeng-070909-105314 20415587

[B69] Sun Y. M. Wang W. T. Zeng Z. C. Chen T. Q. Han C. Pan Q. (2019). circMYBL2, a circRNA from MYBL2, regulates FLT3 translation by recruiting PTBP1 to promote FLT3-ITD AML progression. Blood 134, 1533–1546. 10.1182/blood.2019000802 31387917 PMC6839953

[B70] Suzuki T. (2021). The expanding world of tRNA modifications and their disease relevance. Nat. Rev. Mol. Cell Biol. 22, 375–392. 10.1038/s41580-021-00342-0 33658722

[B71] Tavares J. F. Davis N. K. Poim A. Reis A. Kellner S. Sousa I. (2021). tRNA-modifying enzyme mutations induce codon-specific mistranslation and protein aggregation in yeast. RNA Biol. 18, 563–575. 10.1080/15476286.2020.1819671 32893724 PMC7971265

[B72] Tegowski M. Flamand M. N. Meyer K. D. (2022). scDART-seq reveals distinct m6A signatures and mRNA methylation heterogeneity in single cells. Mol. Cell 82, 868–878.e10. 10.1016/j.molcel.2021.12.038 35081365 PMC8857065

[B73] Tegowski M. Meyer K. D. (2022). Detection of m6A in single cultured cells using scDART-seq. Star. Protoc. 3, 101646. 10.1016/j.xpro.2022.101646 36042888 PMC9420395

[B74] Till J. E. McCulloch E. A. (2011). A direct measurement of the radiation sensitivity of normal mouse. Bone Marrow Cells1 149, 145. 10.1667/RRXX28.1175 21268707

[B75] Tritschler F. Huntzinger E. Izaurralde E. (2010). Role of GW182 proteins and PABPC1 in the miRNA pathway: A sense of déjà vu. Nat. Rev. Mol. Cell Biol. 11, 379–384. 10.1038/nrm2885 20379206

[B76] Trusler O. Huang Z. Goodwin J. Laslett A. L. (2018). Cell surface markers for the identification and study of human naive pluripotent stem cells. Stem Cell Res. 26, 36–43. 10.1016/j.scr.2017.11.017 29227830

[B77] van der Werf J. Chin C. V. Fleming N. I. (2021). Snorna in cancer progression, metastasis and immunotherapy response. Biol. (Basel). 10, 809. 10.3390/biology10080809 PMC8389557 34440039

[B78] Van Niel G. D’Angelo G. Raposo G. (2018). Shedding light on the cell biology of extracellular vesicles. Nat. Rev. Mol. Cell Biol. 19, 213–228. 10.1038/nrm.2017.125 29339798

[B79] Veneziano D. Tomasello L. Balatti V. Palamarchuk A. Rassenti L. Z. Kipps T. J. (2019). Dysregulation of different classes of tRNA fragments in chronic lymphocytic leukemia. Proc. Natl. Acad. Sci. U. S. A. 116, 24252–24258. 10.1073/pnas.1913695116 31723042 PMC6883801

[B80] Verbeek M. W. C. Erkeland S. J. van der Velden V. H. (2022). Dysregulation of small nucleolar RNAs in B-cell malignancies. Biomedicines 10, 1229. 10.3390/biomedicines10061229 35740251 PMC9219770

[B81] Wang Z. Zhang C. Warden C. D. Liu Z. Yuan Y. C. Guo C. (2022). Loss of SIRT1 inhibits hematopoietic stem cell aging and age-dependent mixed phenotype acute leukemia. Commun. Biol. 5, 396. 10.1038/s42003-022-03340-w 35484199 PMC9051098

[B82] Warner W. A. Spencer D. H. Trissal M. White B. S. Helton N. Ley T. J. (2018). Expression profiling of snoRNAs in normal hematopoiesis and AML. Blood Adv. 2, 151–163. 10.1182/bloodadvances.2017006668 29365324 PMC5787869

[B83] Warner W. A. Spencer D. Trissal M. Helton N. Ley T. J. Link D. C. (2015). Characterization of snoRNA expression in acute myeloid leukemia. Blood 126, 3649. 10.1182/blood.v126.23.3649.3649

[B84] Wilkinson A. C. Igarashi K. J. Nakauchi H. (2020). Haematopoietic stem cell self-renewal *in vivo* and *ex vivo* . Nat. Rev. Genet. 21, 541–554. 10.1038/s41576-020-0241-0 32467607 PMC7894993

[B85] Wilkinson K. A. Merino E. J. Weeks K. M. (2006). Selective 2’-hydroxyl acylation analyzed by primer extension (SHAPE): Quantitative RNA structure analysis at single nucleotide resolution. Nat. Protoc. 1, 1610–1616. 10.1038/nprot.2006.249 17406453

[B86] Williams G. T. Farzaneh F. (2012). Are snoRNAs and snoRNA host genes new players in cancer? Nat. Rev. Cancer 12, 84–88. 10.1038/nrc3195 22257949

[B87] Wilson R. C. Doudna J. A. (2013). Molecular mechanisms of RNA interference. Annu. Rev. Biophys. 42, 217–239. 10.1146/annurev-biophys-083012-130404 23654304 PMC5895182

[B88] Wu K. Xing F. Wu S. Y. Watabe K. (2017), Extracellular vesicles as emerging targets in cancer: Recent development from bench to bedside. Biochim. Biophys. acta. Rev. cancer 1868, 538–563. 10.1016/j.bbcan.2017.10.001 29054476 PMC5675795

[B89] Yamauchi T. Masuda T. Canver M. C. Seiler M. Semba Y. Shboul M. (2018). Genome-wide CRISPR-cas9 screen identifies leukemia-specific dependence on a pre-mRNA metabolic pathway regulated by DCPS. Cancer Cell 33, 386–400.e5. 10.1016/j.ccell.2018.01.012 29478914 PMC5849534

[B90] Yao L. Yin H. Hong M. Wang Y. Yu T. Teng Y. (2021). RNA methylation in hematological malignancies and its interactions with other epigenetic modifications. Leuk 355 35, 1243–1257. 10.1038/s41375-021-01225-1 PMC8102199 33767371

[B91] Yin R. Chang J. Li Y. Gao Z. Qiu Q. Wang Q. (2022). Differential m(6)A RNA landscapes across hematopoiesis reveal a role for IGF2BP2 in preserving hematopoietic stem cell function. Cell Stem Cell 29, 149–159.e7. 10.1016/j.stem.2021.09.014 34678169

[B92] Zeleznik O. A. Eliassen A. H. Kraft P. Poole E. M. Rosner B. A. Jeanfavre S. (2020). A prospective analysis of circulating plasma metabolites associated with ovarian cancer risk. Cancer Res. 80, 1357–1367. 10.1158/0008-5472.CAN-19-2567 31969373 PMC7073287

[B93] Zhang C. Chen Y. Sun B. Wang L. Yang Y. Ma D. (2017). m6A modulates haematopoietic stem and progenitor cell specification. Nat. 549, 273–276. 10.1038/nature23883 28869969

[B94] Zhang J. Supakorndej T. Krambs J. R. Rao M. Abou-Ezzi G. Ye R. Y. (2019). Bone marrow dendritic cells regulate hematopoietic stem/progenitor cell trafficking. J. Clin. Invest. 129, 2920–2931. 10.1172/JCI124829 31039135 PMC6597218

[B95] Zhang Z. Chen T. Chen H. X. Xie Y. Y. Chen L. Q. Zhao Y. L. (2021). Systematic calibration of epitranscriptomic maps using a synthetic modification-free RNA library. Nat. Methods 18, 1213–1222. 10.1038/s41592-021-01280-7 34594034

[B96] Zhao C. Zhao Y. Zhao J. Meng G. Huang S. Liu Y. (2022). Acute myeloid leukemia cell-derived extracellular vesicles carrying microRNA-548ac regulate hematopoietic function via the TRIM28/STAT3 pathway. Cancer Gene Ther. 29, 918–929. 10.1038/s41417-021-00378-6 34453123

[B97] Zhou F. Aroua N. Liu Y. Rohde C. Cheng J. Wirth A. K. (2023). A dynamic rRNA ribomethylome drives stemness in acute myeloid leukemia. Cancer Discov. 13, 332–347. 10.1158/2159-8290.CD-22-0210 36259929 PMC9900322

[B98] Zhu Y. Pirnie S. P. Carmichael G. G. (2017). High-throughput and site-specific identification of 2′-O-methylation sites using ribose oxidation sequencing (RibOxi-seq). RNA 23, 1303–1314. 10.1261/rna.061549.117 28495677 PMC5513074

